# Highly compressible 3D periodic graphene aerogel microlattices

**DOI:** 10.1038/ncomms7962

**Published:** 2015-04-22

**Authors:** Cheng Zhu, T. Yong-Jin Han, Eric B. Duoss, Alexandra M. Golobic, Joshua D. Kuntz, Christopher M. Spadaccini, Marcus A. Worsley

**Affiliations:** 1 Lawrence Livermore National Laboratory, 7000 East Avenue, Livermore, California 94550, USA

## Abstract

Graphene is a two-dimensional material that offers a unique combination of low density, exceptional mechanical properties, large surface area and excellent electrical conductivity. Recent progress has produced bulk 3D assemblies of graphene, such as graphene aerogels, but they possess purely stochastic porous networks, which limit their performance compared with the potential of an engineered architecture. Here we report the fabrication of periodic graphene aerogel microlattices, possessing an engineered architecture via a 3D printing technique known as direct ink writing. The 3D printed graphene aerogels are lightweight, highly conductive and exhibit supercompressibility (up to 90% compressive strain). Moreover, the Young's moduli of the 3D printed graphene aerogels show an order of magnitude improvement over bulk graphene materials with comparable geometric density and possess large surface areas. Adapting the 3D printing technique to graphene aerogels realizes the possibility of fabricating a myriad of complex aerogel architectures for a broad range of applications.

## Abstract (web summary)

Aerogels are ultra-lightweight porous materials that possess some remarkable properties. Here, the authors use a 3D printing technique to fabricate just such a material out of graphene, exhibiting large surface area, high conductivity and supercompressibility while maintaining good structural integrity.

## Graphical abstract

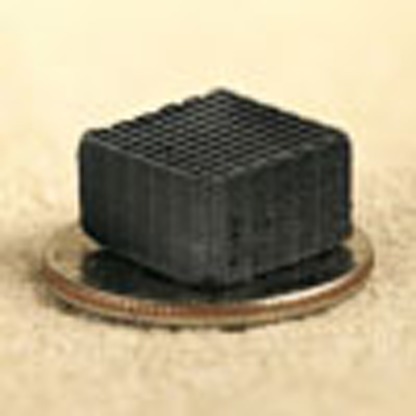

Graphene is an emerging class of ultrathin carbon membrane material^1^,^2^,^3^ with high specific surface area^4^, superior elasticity^5^, chemical stability^3^ and high electrical and thermal conductivity^6^,^7^. These intrinsic physicochemical properties enable graphene to find widespread applications in nanoelectronics^8^,^9^, sensors^10^,^11^, catalysis^12^,^13^, composites^14^,^15^, energy storage^16^,^17^ and biomedical scaffolds^18^. To further explore various macroscopic applications of graphene materials, an essential prerequisite is controlled large-scale assembly of two-dimensional graphene building blocks and transfer of their inherent properties into three-dimensional (3D) structures. Template-guided methods, such as chemical vapour deposition coatings on metallic foams^19^ have been reported for the creation of 3D graphene monoliths, but the process is not scalable and the materials obtained from these methods are generally brittle under low compression^20^. Therefore, template-free approaches are still needed for scalable synthesis of 3D graphene macro-assemblies. Due to their simple and versatile synthesis scheme, and the ability to realize a wide range of pore morphologies, including ultrafine pore sizes (<100 nm), chemically derived graphene oxide (GO)-based aerogels are the most common 3D graphene found in the literature^20^,^21^,^22^,^23^,^24^,^25^,^26^,^27^,^28^,^29^,^30^,^31^,^32^,^33^,^34^,^35^,^36^. Starting from a widely available GO precursor, the main strategy to assemble porous 3D graphene networks is the self-assembly or gelation of the GO suspension via hydrothermal reduction^20^,^21^,^22^, chemical reduction^27^,^28^,^29^,^30^,^31^ or direct crosslinking^33^,^34^ of the GO sheets. Although some control over the pore morphology has been demonstrated with ice templating^35^,^36^, the architecture of these graphene networks remains largely random, precluding the ability to tailor transport and other mechanical properties of the material for specific applications (for example, separations, flow batteries, pressure sensors and so on) that might benefit from such engineering. Thus the fabrication of 3D graphene materials with tailored macro-architectures for specific applications via a controllable and scalable assembly method remains a significant challenge.

The functional characteristics of cellular materials are mostly determined by the intrinsic properties of their chemical composition, porosity and cell morphologies^37^. Several additive manufacturing techniques have been utilized to make highly ordered ultralight cellular materials possessing unique chemical, mechanical and structural properties by manipulation of their structures from the nanometre up to the centimetre scale^38^. For example, ultralight hollow metallic microlattices were produced using self-propagating photopolymer waveguide prototyping to form a template and subsequently coating the template with nickel–phosphorus via electroless plating^39^. Another example is the fabrication of ultralight, ultra-stiff octet truss metamaterials by the projection micro-stereolithography method^40^. However, these methods have limitations in their scaling and material diversity. Recently, an extrusion-based 3D printing technique, known as direct ink writing has also been applied to construct cellular elastomeric architectures^41^ and lightweight composites^42^. This technique employs a three-axis motion stage to assemble 3D structures by robotically extruding a continuous ‘ink' filament through a micronozzle at room temperature in a layer-by-layer scheme^43^. The primary challenge for this method is to design gel-based viscoelastic ink materials possessing shear thinning behaviour to facilitate flow under pressure and a rapid pseudoplastic to dilatant recovery after deposition resulting in shape retention^44^,^45^. Although a number of ceramic, metallic, polymeric and even graphene–polymer composite ink materials^43^,^46^,^47^ have been developed to fabricate various complex 3D structures, there is no example using this technique to create 3D periodic graphene aerogel macro-architectures.

In this work, we demonstrate a 3D printing strategy for the fabrication of 3D graphene aerogels with designed macroscopic architectures. Our approach is based on the precise deposition of GO ink filaments on a pre-defined tool path to create architected 3D structures. Two key challenges in this process are developing a printable graphene-based ink and maintaining the intrinsic properties of single graphene sheets (for example, large surface area, mechanical and electrical properties) in the 3D printed structures. To this end, we have developed a new GO-based ink and printing scheme that allows the manufacture of porosity-tunable hierarchical graphene aerogels with high surface area, excellent electrical conductivity, mechanical stiffness and supercompressibility.

## Results

### Three-dimensional printing of graphene aerogels

**Figure 1:**
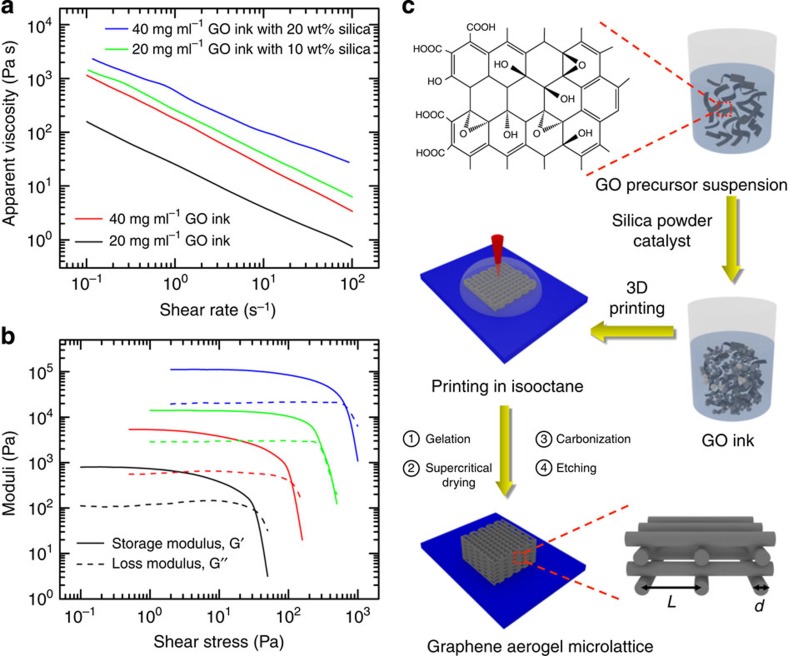
Fabrication strategy and GO ink's rheological properties. Log–log plots of (**a**) apparent viscosity as a function of shear rate and (**b**) storage and loss modulus as a function of shear stress of GO inks with and without silica fillers. (**c**) Schematic of the fabrication process. Following the arrows: fumed silica powders and catalyst (that is, (NH_4_)_2_CO_3_ or R–F solution) were added into as-prepared aqueous GO suspensions. After mixing, a homogeneous GO ink with designed rheological properties was obtained. The GO ink was extruded through a micronozzle immersed in isooctane to prevent drying during printing. The printed microlattice structure was supercritically dried to remove the liquid. Then, the structure was heated to 1,050 °C under N_2_ for carbonization. Finally, the silica filler was etched using HF acid. The in-plane centre-to-centre rod spacing is defined as *L*, and the filament diameter is defined as *d*.

The first challenge for this fabrication strategy is to develop printable GO inks, by tailoring the composition and rheology required for reliable flow through fine nozzles, and self-supporting shape integrity after deposition (for example, highly viscous, non-Newtonian fluids). Printable GO ink development is challenging because most GO-based graphene aerogels begin with fairly dilute precursor GO suspensions (<5 mg ml^−1^ GO) that do not possess the required rheological behaviour for a 3D printable ink as they are low-viscosity (*η*) Newtonian fluids^20^,^48^. Recently, the rheological behaviour of GO dispersions has been investigated to enable further fabrication of GO into complex architectures^49^. There are reports of higher concentration GO suspensions (for example, 10–20 mg ml^−1^ GO) that can also make high-quality graphene aerogels^30^,^33^. These reports demonstrate gelation of concentrated GO suspensions under basic conditions (for example, addition of ammonium hydroxide) or direct crosslinking using organic sol–gel chemistry (for example, resorcinol–formaldehyde (R–F) solution). As the gelation method can influence the aerogel microstructure^34^, both methods were applied to the high-concentration GO suspensions we investigated for the GO inks. Figure 1a shows the apparent viscosity of high-concentration GO suspensions as a function of shear rate, revealing that at 20 mg ml^−1^, the GO suspension shows orders of magnitude higher apparent viscosity than reported at lower concentrations^48^, and that the GO suspension at 20 mg ml^−1^ is a shear-thinning non-Newtonian fluid, which is necessary for a printable ink. Further increasing the GO concentration to 40 mg ml^−1^ results in another order-of-magnitude increase in apparent viscosity, which further improves printability. Finally, addition of hydrophilic fumed silica powders to the GO suspensions imparts additional increases in viscosity. Silica filler serves as a removable viscosifier by imparting both shear thinning behaviour and a shear yield stress to the GO suspension to further enhance the printability of the GO inks. Figure 1b compares the pure GO suspensions and representative GO inks storage and loss moduli with varying compositions. Specifically, the pure 20-mg ml^−1^ GO suspensions without fillers exhibit a plateau value of its elastic modulus (*G′*) ∼1,000 Pa and a yield stress (*τ*_*y*_) ∼40 Pa, respectively. By adding 20 wt% silica powders into pure 20 mg ml^−1^ GO suspensions, both elastic modulus and yield stress increase by approximately an order of magnitude. Meanwhile, the addition of 10 wt% silica filler increases the elastic modulus and yield stress of 40 mg ml^−1^ GO suspensions by over an order magnitude. The magnitudes of these key rheological parameters are in good agreement with those reported for other colloidal inks designed for this 3D filamentary printing technique^45^. Although the pure 40-mg ml^−1^ GO suspension ink is printable, the silica-filled GO inks were preferred due to their superior rheological properties and facile removal of the silica during post-processing. In addition to these, GO inks exhibit the desired viscoelasticity and they have a long pot life.

The process of 3D printing the GO inks such that a 3D graphene aerogel structure is produced also presents several obstacles. Aerogels are ultralow-density porous solids created by carefully replacing the liquid in the pores of the wet gel with air. To convert the 3D printed GO structure to an aerogel, the GO ink must remain wet through printing and gelation such that the liquid in the GO gel can be removed via supercritical- or freeze-drying to avoid gel collapse due to capillary forces. This necessitates printing the GO ink into a bath of liquid that is not only less dense than water but immiscible with our aqueous GO inks. The fabrication scheme for accomplishing this is illustrated in Fig. 1c. An animation of the fabrication scheme used to print the graphene aerogel microlattices can also be seen in Supplementary Movie 1. The GO inks are prepared by combining a GO suspension and silica filler to form a homogenous, highly viscous and thixotropic ink. These GO inks are then loaded into a syringe barrel and extruded through a micronozzle to pattern 3D structures. To prevent the ink from drying in the air, which can clog the tip of the printing apparatus or cause pore collapse in the printed structure, the printing is carried out in an organic solvent bath (isooctane) that is not miscible with the aqueous ink. Finally, the printed structures can be processed according to standard literature methods^29^,^30^, followed by etching of the silica filler to obtain the ultimate periodic 3D graphene aerogel microlattices.

**Figure 2:**
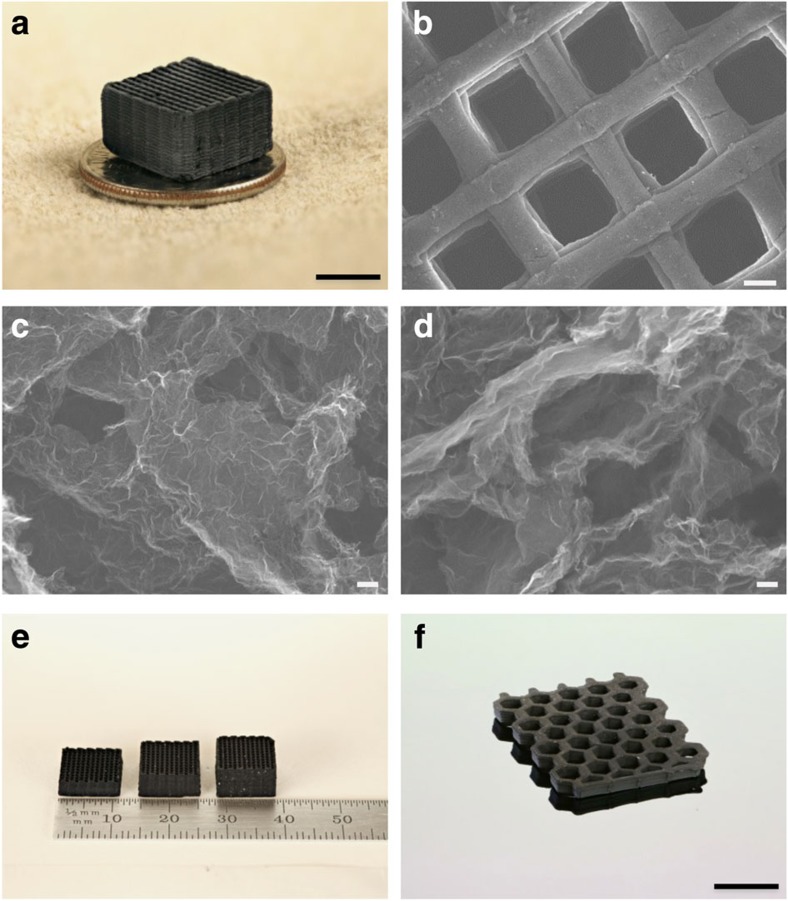
Morphology and structure of graphene aerogels. (**a**) Optical image of a 3D printed graphene aerogel microlattice. SEM images of (**b**) a 3D printed graphene aerogel microlattice, (**c**) graphene aerogel without R–F after etching and (**d**) graphene aerogel with 4 wt% R–F after etching. Optical image of (**e**) 3D printed graphene aerogel microlattices with varying thickness and (**f**) a 3D printed graphene aerogel honeycomb. Scale bars, 5 mm (**a**), 200 μm (**b**), 100 nm (**c**,**d**), 1 cm (**f**).

To demonstrate 3D printing of graphene aerogels, we first printed woodpile, ‘simple cubic'-like lattices consisting of multiple orthogonal layers of parallel cylindrical filaments successively printed in a layer-by-layer fashion. These 3D simple cubic lattices are designed with an in-plane centre-to-centre filament spacing (*L*) of 1 mm and a filament diameter (*d*) of 0.25 mm, resulting in a spacing-to-diameter ratio (*L/d*) of 4 (Fig. 1c). By simply changing the filament spacing and diameter, we have the ability to 3D print graphene structures over a wide range of geometric densities. The printed 3D graphene aerogel microlattice shows excellent structural integrity and micro-architecture accuracy (Fig. 2a,b), which is indicative of the high quality of the ink material for this printing process (see Supplementary Movie 2). After the removal of silica fillers (Supplementary Fig. 1a), there are random large pores distributed in graphene aerogels (Fig. 2c,d; Supplementary Fig. 1b). Figure 2c,d also shows how the microstructure of the 3D printed graphene aerogels can be tuned by simply modifying the GO ink formulations. Similar to results observed in bulk monolithic graphene aerogels^34^, changes in the gelation chemistry can lead to significant microstructural changes. In this case, we use either basic solution (for example, (NH_4_)_2_CO_3_) to directly crosslink graphene sheets via the functional groups (for example, epoxide and hydroxide) or resorcinol (R) and formaldehyde (F) with sodium carbonate as a catalyst to ‘glue' the sheets together. The use of organic sol–gel chemistry (R–F solution) to build the GO network led to a more open, less crosslinked network (Fig. 2d) compared with gelation methods based on GO's native functionality (that is, no R–F) (Fig. 2c). The ability to tune the microstructure, in addition to the macrostructure, is important because it can affect a wide range of properties such as density, conductivity, surface area and, as noted below, mechanical properties. This approach opens new opportunities for the fabrication of graphene-based structures at the macroscale. To further demonstrate the flexibility of this 3D printing technique, we fabricated a series of graphene aerogel microlattices with varying thicknesses and a large area graphene aerogel honeycomb (Fig. 2e,f).

### Physical properties of 3D printed graphene aerogels

**Figure 3:**
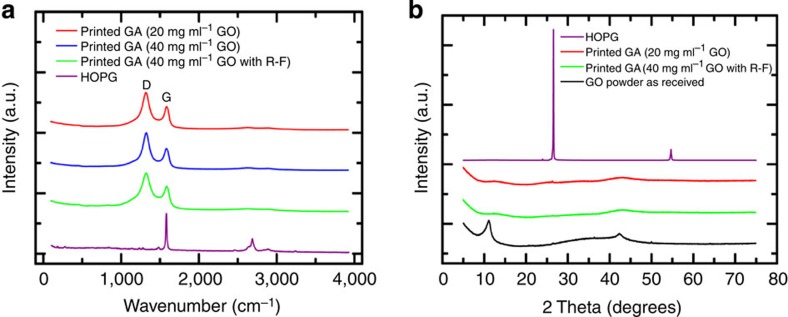
Raman and XRD spectra of graphene aerogels. (**a**) Raman and (**b**) XRD spectra of 3D printed graphene aerogel microlattices made with various ink formulations. Spectra of highly oriented pyrolytic graphite (HOPG) and graphene oxide (GO) powder are included for reference.

Modifying the GO suspensions to make suitable inks has the potential to alter the properties of the final aerogel; however, most properties of the 3D printed graphene aerogels were found to meet or exceed those of the bulk material. For example, techniques such as Raman spectroscopy, X-ray diffraction (XRD) and energy-dispersive X-ray spectroscopy (EDS) were applied to see how microstructure, graphene layering and degree of GO reduction compare with bulk graphene aerogels. Raman spectra of the 3D printed graphene aerogels (Fig. 3a) all show strong D (1,350 cm^−1^) and G (1,582 cm^−1^) bands with weak, broad D′ and G′ features identical to those previously reported for bulk aerogels^29^,^30^, suggesting a similar microstructure and defect density. XRD spectra of 3D printed graphene aerogels (Fig. 3b) are also similar to those of bulk graphene aerogels^29^,^30^, showing weak, broad features indicative of single- and few-layer graphene. EDS (Supplementary Fig. 2) also shows that, like the bulk graphene aerogel, the C:O ratio of 3D printed graphene aerogel rises to >20 compared with 5 for the native GO, confirming a high level of GO reduction. EDS also confirms that the silica filler has been completely removed from the graphene microlattice. Together, the scanning electron microscopy (SEM), Raman, XRD and EDS show that the 3D printed graphene aerogel is quite similar to the bulk graphene aerogel and is not significantly degraded by the etching or printing process.

Standard graphene aerogels are also notable for their large surface areas, low densities and high electrical conductivities. These characteristics are also evaluated for the modified formulations that we used to create the inks and are presented in Table 1. Nitrogen porosimetry (Table 1; Supplementary Fig. 3) show that the modified formulations maintain a high surface area (700–1,100 m^2^ g^−1^) and large mesopore volumes (2–4 cm^3^ g^−1^), consistent with the SEM images and comparable to bulk graphene aerogels in the literature^29^,^30^. Four-probe and density measurements also show that the modified formulations retain a low density and high conductivity characteristic of standard graphene aerogels. As seen in previous reports^29^,^30^, all these properties (surface area, conductivity and density) can be tuned by changing the R–F concentration in the initial suspension. The GO concentration also appears to impact the surface area of the aerogel. The slightly lower surface areas at higher GO concentrations likely stem from larger fractions of few-layer graphene due to less efficient exfoliation.

**Figure 4:**
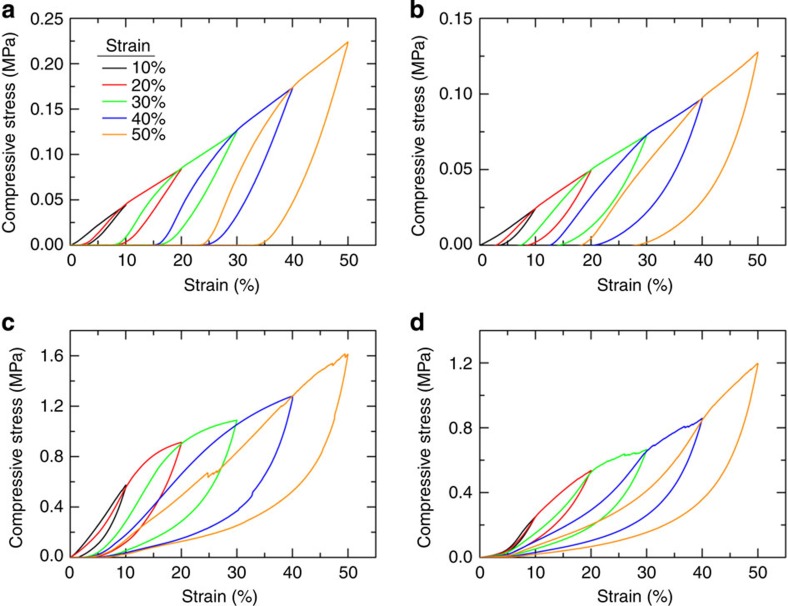
Compressive properties of graphene aerogels. Stress–strain curves during loading–unloading cycles in sequence of increasing strain amplitude for (**a**) bulk graphene aerogel (31 mg cm^−3^) and (**b**) 3D printed graphene aerogel microlattice (16 mg cm^−3^) using the GO ink without R–F, (**c**) bulk graphene aerogel (123 mg cm^−3^) and (**d**) 3D printed graphene aerogel microlattice (53 mg cm^−3^) using the GO ink with R–F.

Graphene aerogels are also known to be remarkably stiff and flexible. To quantify the mechanical properties of these aerogels, we conducted in-plane compression tests to measure the compressive stress (*σ*) as a function of strain (*ɛ*) for all bulk and printed structures. The compressibility of these graphene aerogels is displayed in Fig. 4. It presents the stress–strain curves of five stepped compression cycles with strain amplitude of 10, 20, 30, 40 and 50% in sequence. The starting point for each cycle is the same and equal to the initial thickness of the sample, no matter how much unrecoverable compression is in the previous cycle. It is interesting that each succeeding loading curve exactly rises back to the maximum stress–strain point of the preceding cycle and continues the trend of the preceding loading curve in the full range of our measurements, showing a perfect strain memory effect. Figure 4a,b shows the stress–strain curves of bulk and printed graphene aerogels using the native functionality of the GO sheets with loading curves that display linear elastic properties from 10 to 50% strain. From the unloading curves, we can find each compression leads to a degree of permanent residual deformation, and the recoverability of the printed aerogels is slightly higher than that of bulk aerogels.

In contrast, bulk and printed graphene aerogels using GO inks with organic sol–gel chemistry to crosslink GO sheets exhibit extraordinary supercompressibility, with full recovery after large strains (Fig. 4c,d). As the main difference between the aerogels lies in their microstructure (Fig. 2c,d)^34^, we propose that the difference in compressive behaviour is linked to their microstructural differences. The loading curves of both bulk and printed aerogels show three distinct regions typically observed in other cellular materials, namely an initial Hookean region at 5%<*ɛ*<10%, a plateau at 10%<*ɛ*<40% and a densification regime for *ɛ*>40% with a steep increase in stress. Thus, similar to other resilient cellular materials^36^,^37^, hysteresis loops are found in the loading–unloading cycles, which indicate energy dissipation that can be attributed to the buckling of microstructures, the friction and adhesion between branches and the cracks that occur primarily in the first compression for the large dissipation. The initial increase of stress in the range of *ɛ*<5% is attributed to the increase of contact area between the sample and the platen for our compression fixture. The primary deformation in the Hookean region is linear elastic dominated by bending mode deformation. The plateau is mainly attributed to the buckling deformation of the graphene sheets. As the graphene aerogel crosslinked via organic sol–gel chemistry has a more open, less crosslinked microstructure and the graphene sheets are free to bend and buckle under compression, there is substantial recovery when the load is removed. Even after compression to 90% strain, less than 5% residual deformation was observed (Supplementary Fig. 4).

**Figure 5:**
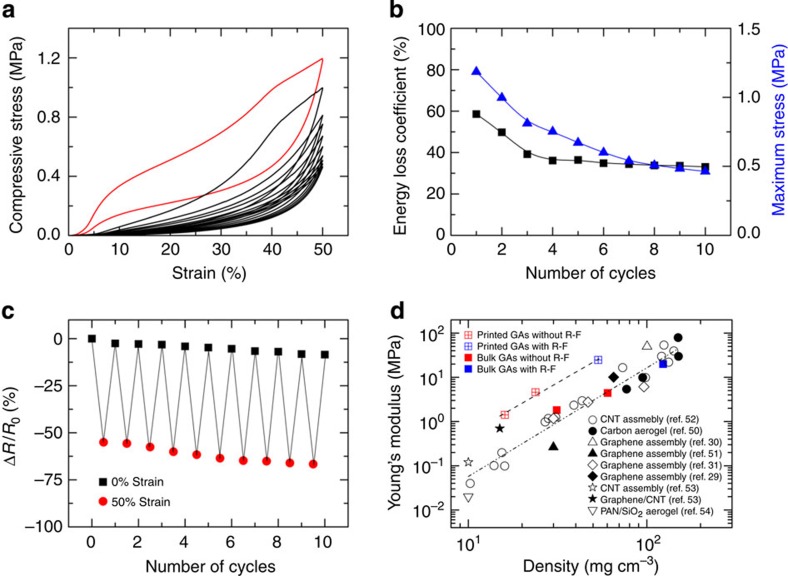
Physical properties of graphene aerogels. (**a**) Compressive stress–strain curves of 10 cycles of loading–unloading. (**b**) Maximum stress and energy loss coefficient during 10 cycles. (**c**) Electrical resistance change when repeatedly compressed up to 50% of strain for 10 cycles. The graphene aerogel microlattice used for cyclic compression and conductivity measurements (**a**–**c**) has a geometric density of 53 mg cm^−3^. (**d**) The relationships between Young's modulus and density of bulk and printed graphene aerogels.

To further assess and characterize the stability of the cyclic resilient property of printed graphene aerogels, compression cycling of the graphene aerogel at 50% strain was conducted (Fig. 5). Energy dissipation is one of the key functions of cellular materials, and our printed graphene elastomers exhibit excellent energy absorption capability. In Fig. 5a,b, the energy loss coefficient of printed aerogels decreases from 60 to 30% in the first three cycles and then remains fairly constant. The maximum stress for each cycle in Fig. 5a also shows a similar trend (Fig. 5b). The electrical resistance of the printed graphene aerogels was also determined as a function of cyclic compression (Fig. 5c). The electrical resistance of the printed aerogels shows only a slight decrease after multiple compression events, confirming the remarkable structural resilience of the graphene aerogel microlattices.

Finally, the effect of macroscopic architectural design on the mechanical properties is also reflected in superior rigidity of the graphene aerogel microlattices compared with bulk aerogels at the same overall geometric density. It has been shown that the stiffness of many conventional cellular solids is significantly diminished as their densities decrease due to quadratic or higher power scaling relationships between Young's modulus (*E*) and density (*ρ*)^37^. Figure 5d shows the Young's modulus as a function of density for our graphene microlattices (printed) and standard graphene aerogels (bulk) compared against other carbon, carbon nanotube and graphene assemblies found in the literature^29^,^30^,^31^,^50^,^51^,^52^,^53^,^54^ as a function of density. The bulk aerogel data are consistent with literature data, while the printed aerogel data are substantially offset from the known curve. The log–log plot in each case demonstrates the expected power-law density dependence of Young's modulus. In both cases, Young's modulus was found to scale with density as *E*∝*ρ*^2.5^, consistent with other studies^29^. The similar values of the exponent (∼2.5) indicate both printed and bulk aerogels show the same bending-dominated behaviour under compression. However, the magnitude of *E* for the graphene microlattices was about one order of magnitude larger than that of most bulk graphene aerogels with same densities. In other words, the printed graphene aerogels can maintain the stiffness values of higher-density bulk aerogels to much lower densities. This phenomenon is also commonly observed in traditional cellular materials, such as honeycombs, which exhibit stiffness values in certain loading directions that rival higher-density bulk solids^37^. In the case of the printed aerogels, the printed structures exhibit Young's moduli that rival bulk aerogel values with nearly twice the density of their printed counterparts. Upon closer inspection, it is revealed that the Young's modulus of the printed structure is approximately equal to that of the bulk aerogel with the same density as that of the printed aerogel filaments within the lattice. Thus, the improved performance is primarily attributed to the local density in the printed aerogels rather than the overall density, which accounts for the macroscale pores. In other words, the stiffness is controlled by the density of each filament, which is much higher than the geometric density of total microlattice. In fact, the actual geometric density of the printed aerogel is quite consistent with the theoretically expected value for each lattice (Supplementary Fig. 5). These observations show that the introduction of periodic macroscale pores in the 3D printed microlattices can provide additional functionality to the aerogel (for example, lower density, faster mass transport and so on) with negligible impact on the mechanical integrity of structure. Thus, the 3D printed graphene aerogels would benefit technologies such as catalysis, desalination and other filtration/separation applications that require large surface areas, low density, superior mechanical rigidity and engineered porosity for predictable fluid flow characteristics.

## Discussion

We present a general strategy for fabrication of periodic graphene aerogel microlattices via 3D printing. Key factors for successful 3D printing of aerogels included modifying GO precursor suspension such that it serves as printable ink, and adapting the 3D printing process to prevent premature drying of the printed structure. By addressing these issues, 3D printed aerogel microlattices were produced with properties that met or exceeded those of bulk aerogel materials. The graphene microlattices possess large surface areas, good electrical conductivity, low relative densities and supercompressibility, and are much stiffer than bulk graphene of comparable geometric density. By modifying the microstructure and density of the graphene aerogel through changing the ink formulation, we also showed how mechanical properties of the microlattices can be tuned. As graphene aerogels are currently being explored for a broad range of applications, having a manufacturing method for creating periodic or engineered structures using this novel material will further expand the range of applications where graphene can be utilized. In particular, our strategy makes it possible to explore the properties and applications of graphene in a self-supporting, structurally tunable and 3D macroscopic form. This work presents a versatile method for fabricating a broad class of 3D macroscopic graphene aerogel structures of determined geometries, and could lead to new types of graphene-based electronics, energy storage devices, catalytic scaffolds and separation devices. Furthermore, other functional materials can be readily incorporated into the open void space, offering opportunity to create new graphene-based nanocomposites. Finally, this fabrication scheme could be broadly applied to other aerogel systems enabling 3D printed aerogel structures for the myriad of technologies that require high surface area, low-density materials.

## Methods

### GO ink preparation

Raw single-layer GO powder (Cheaptubes) was produced by the Hummer method^55^ and had lateral dimensions of 300–700 nm. GO suspensions were prepared via ultrasonication at concentrations of 20 and 40 mg ml^−1^ in water for 24 h in a VWR Scientific Model 57T Aquasonic (sonic power∼90 W, frequency∼40 kHz). After sonication, the lateral dimensions were in the range of 150–400 nm. For GO inks gelled using the native functionality on the GO sheets, ammonium carbonate ((NH_4_)_2_CO_3_) solution (0.3 wt%) was used. In a typical synthesis, GO inks are prepared by mixing 6 g of 40 mg ml^−1^ GO suspension, 0.343 g (NH_4_)_2_CO_3_ solution and 0.7 g fumed silica (EH-5, Cabot), respectively. For GO inks gelled using organic sol–gel chemistry, the sol–gel mixture consisted of an aqueous solution of resorcinol (R), formaldehyde (F) and sodium carbonate catalyst (C). The R:F mole ratio was 1:2, the R:C mole ratio was 200:1 and the resultant R–F concentration was 11 wt% R–F solids. In a typical synthesis, 3.6 g of 40 mg ml^−1^ GO suspensions, 2 g of R–F solution and 0.7 g of fumed silica are mixed. A planetary centrifugal mixer (Thinky) was used for mixing these samples for 2 min in a 35-ml container using a custom adaptor.

### Ink rheology

Rheological properties of the inks were characterized using a stress-controlled Rheometer (AR 2000ex, TA) with a 40-mm-flat plate geometry and a gap of 500 μm. All measurements were preceded by a 1-min conditioning step at a constant shear rate of 1 s^−1^, followed by a 10-min rest period to allow the ink structure to reform. A stress sweep from 10^−2^ to 10^3^ Pa at a constant frequency of 1 Hz was conducted to record the storage modulus and loss modulus variations as a function of sweep stress. The yield stress (*τ*_*y*_) was defined as the stress where storage modulus falls to 90% of the plateau value. A strain sweep from 10^−1^ to 10^2^ s^−1^ was also performed to record the apparent viscosity at varying shear rates.

### 3D printing

The GO ink was housed in a 3 ml syringe barrel (EFD) attached by a luer-lok to a smooth-flow tapered nozzle (250 μm inner diameter, *d*). An air-powered fluid dispenser (Ultimus V, EFD) provided the appropriate pressure to extrude the ink through the nozzle. The target patterns were printed using a three-axis positioning stage (ABL 9000, Aerotech The 3D GO structures were printed onto silicon wafers in an isooctane (2,2,4-trimethylpentane) bath, with an initial nozzle height of 0.7*d* to ensure adhesion to the substrate. Three-dimensional periodic microlattices were assembled by patterning an array of parallel (rod-like) filaments in a meanderline-like pattern in the horizontal plane such that the orientation of each successive layer was orthogonal to the previous layer. Three-dimensional honeycomb structures were fabricated by stacking hexagonal unit-cell arrays into a lattice and then printed directly upon previous layers. Printed parts were cured in sealed glass vials at 85 °C. After gelation, the wet GO gels were removed from the glass vials and washed in acetone to remove water from the pores. Supercritical CO_2_ was used to dry the GO gels, and they were thermally reduced at 1,050 °C under nitrogen. Finally, etching in hydrofluoric acid solution was used to remove the silica nanoparticle filler.

### Characterization

The dimension and weight of the samples were determined with a caliper with an accuracy of 0.01 mm and an ultra-micro balance (XP24, Mettler Toledo) with an accuracy of 0.001 mg. The relative density was calculated from the measured mass and volume of each specimen. The compressive characteristics of printed specimens were measured using a universal testing machine (Instron 5943) equipped with a 1,000 N load cell at a nominal rate of 5 μm s^−1^. The Young's modulus was calculated from the initial slope of the unloading stress–strain curves between 0 and 10% strain ranges^56^,^57^,^58^. Simultaneously, the electrical conductivity was measured by a two-electrode method and two metal wires were used as the current collectors. To optimize the electrical contact between conductive copper face sheets and aerogel, each end of the aerogel was carefully affixed to copper sheet with a thin layer of silver paste. The morphology of the printed samples was observed by optical camera and field-emission SEM. SEM and EDS was performed on a JEOL 7401-F at 10 keV (20 mA) in secondary electron imaging mode with a working distance of 2–8 mm. Electrical conductivity was measured using the four-probe method. Textural properties were determined by Brunauer–Emmett–Teller and Barrett–Joyner–Halenda methods using an ASAP 2000 Surface Area Analyzer (Micromeritics Instrument Corporation) via nitrogen porosimetry^1^. Samples of ∼0.1 g were heated to 150 °C under vacuum (10^−5^ Torr) for at least 24 h to remove all adsorbed species. XRD measurements were performed on a Bruker AXS D8 ADVANCE X-ray diffractometer equipped with a LynxEye 1-dimensional linear Si strip detector. The samples were scanned from 5 to 75° 2*θ*. The step scan parameters were 0.02° steps and 2 s counting time per step with a 0.499° divergence slit and a 0.499° antiscatter slit. The X-ray source was Ni-filtered Cu radiation from a sealed tube operated at 40 kV and 40 mA. Phases in the samples were identified by comparison of observed peaks to those in the International Centre for Diffraction Data (ICDD PDF2009) powder diffraction database, and also peaks listed in reference articles. Goniometer alignment was ensured using a Bruker-supplied Al_2_O_3_ standard.

## Author contributions

C.Z., E.B.D., J.D.K., C.M.S. and M.A.W. conceived and designed experiments. C.Z., M.A.W., T.Y.-J.H. and A.M.G. prepared samples. M.A.W. and C.Z. were involved in electrical analysis. M.A.W. performed SEM, Raman, XRD, EDS and surface area analysis. C.Z. performed rheological and mechanical experiments. M.A.W., C.Z., E.B.D. and C.M.S. were mainly responsible for preparing the manuscript with further inputs from other authors. All authors discussed the results and commented on the manuscript.

## Additional information

**How to cite this article:** Zhu, C. *et al*. Highly compressible 3D periodic graphene aerogel microlattices. *Nat. Commun*. 6:6962 doi: 10.1038/ncomms7962 (2015).

## Supplementary Material

Supplementary Information Supplementary Figures 1-5.

Supplementary Movie 1 An animation showing the fabrication scheme used to print the graphene aerogel microlattices

Supplementary Movie 2 Real-time video showing the 3D printing of GO ink in 2,2,4- trimethylpentane (isooctane) bath.

## Figures and Tables

**Table 1:** Physical properties of different 3D printed graphene aerogel formulations.

Ink formulation	Aerogel density (mg cm^−3^)	BET surface area (m^2^ g^−1^)	Pore volume (cm^3^ g^−1^)	Conductivity (S m^−1^)
20 mg ml^−1^ GO	31	1,066	4.1	87
40 mg ml^−1^ GO	60	955	3.8	198
40 mg ml^−1^ GO with R–F	123	704	2.5	278

BET, Brunauer–Emmett–Teller; 3D, three-dimensional; GO, graphene oxide; R–F, resorcinol–formaldehyde.
