# Supplementary material for: Highly compressible 3D periodic graphene aerogel microlattices
Source: Nat Commun. 2015 Apr 22;6:6962. doi: 10.1038/ncomms7962 (PMC4421818; doi:10.1038/ncomms7962)
Supplement: Supplementary Information — Supplementary Figures 1-5. [file ncomms7962-s1.pdf]

Supplementary Figures

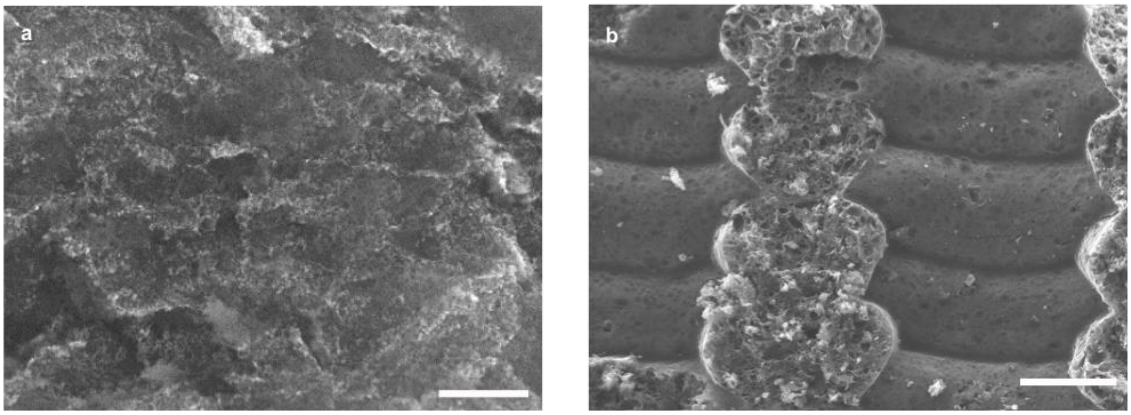

Supplementary Figure 1. (**a**) SEM image of 3D printed graphene before etching and (**b**) after etching the silica nanoparticle filler. Scale bar, (a) 1 μm (b) 200 μm.

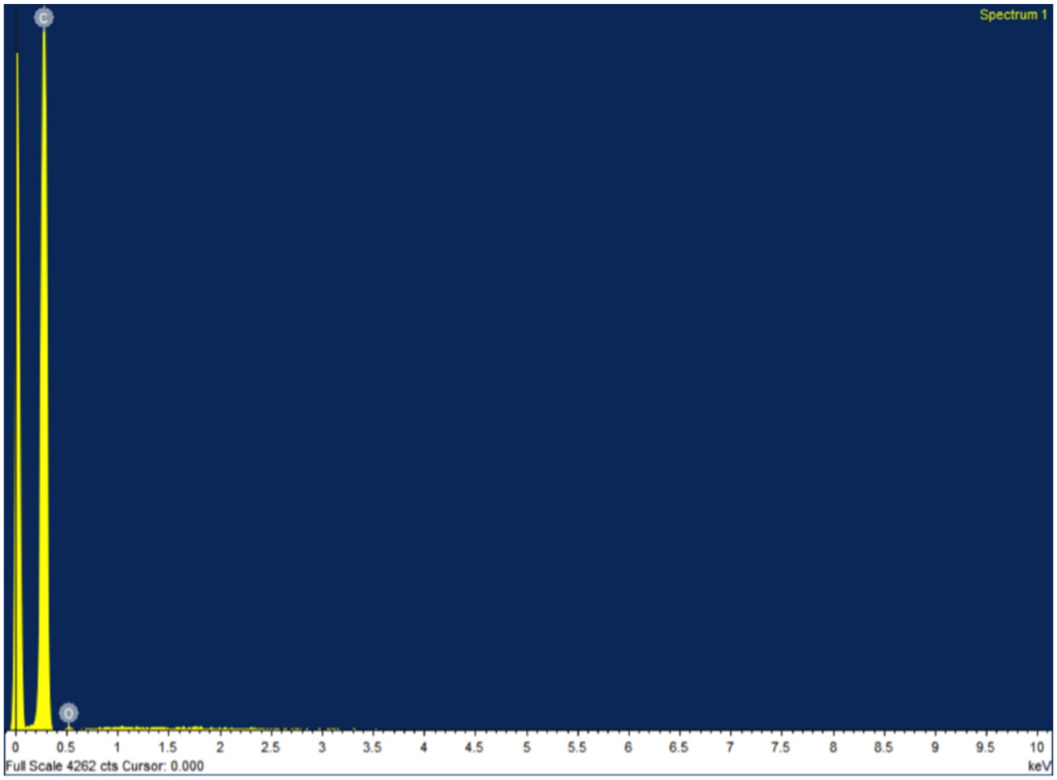

Supplementary Figure 2. EDS spectra of 3D printed graphene aerogel lattice after etching. Carbon content is 98.9 at%. Oxygen content is 1.1 at%. Silicon content was below the detection limit.

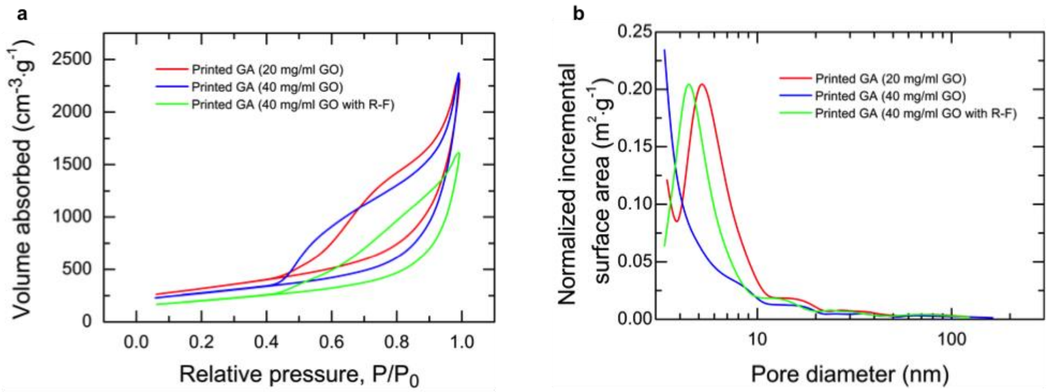

Supplementary Figure 3. (a) Nitrogen adsorption-deposition isotherms and (b) pore size distributions (BJH) for 3D printed graphene aerogels.

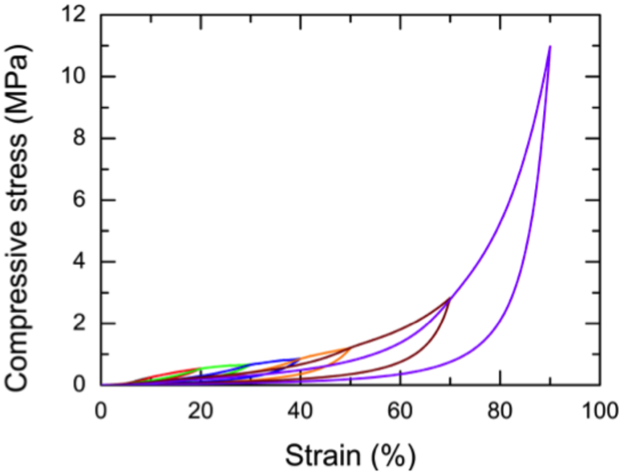

Supplementary Figure 4. Stress-strain curves during loading-unloading cycles in sequence of increasing strain amplitude from 10, 20, 30, 40, 50, 70 to 90% for 3D printed graphene aerogel microlattice with geometric density of 58 $mg \cdot cm^{-3}$.

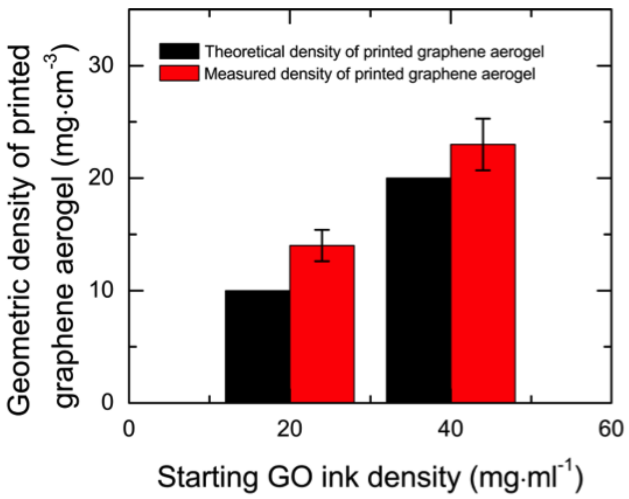

Supplementary Figure 5. Comparison of geometric density of theoretical and measured densities of printed graphene aerogel microlattices from two starting GO inks with varying densities.
